# Secondary metabolites with antimicrobial activity produced by thermophilic bacteria from a high-altitude hydrothermal system

**DOI:** 10.3389/fmicb.2024.1477458

**Published:** 2024-09-30

**Authors:** Coral Pardo-Esté, Johanna Cortés, Juan Castro-Severyn, Vilma Pérez, Karem Henriquez-Aedo, Fabian Cuadros, Carolina Yañez, Sara Cuadros-Orellana, Cristina Dorador, Veronica Molina, Yoanna Eissler, Pablo Paquis, Wade H. Jeffrey, Patricia Pozo, Pablo A. Pérez, Martha B. Hengst

**Affiliations:** ^1^Laboratorio de Ecología Molecular y Microbiología Aplicada, Departamento de Ciencias Farmacéuticas, Facultad de Ciencias, Universidad Católica del Norte, Antofagasta, Chile; ^2^Microbial Ecology of the Rhizosphere Group, Universidad Católica de Valparaíso, Valparaiso, Chile; ^3^Laboratorio de Microbiología Aplicada y Extremófilos, Departamento de Ingeniería Química, Universidad Católica del Norte, Antofagasta, Chile; ^4^Australian Centre for Ancient DNA (ACAD), University of Adelaide, Adelaide, SA, Australia; ^5^Centre of Excellence for Australian Biodiversity and Heritage, University of Adelaide, Adelaide, SA, Australia; ^6^Laboratorio de Biotecnología y Genética de los Alimentos, Departamento de Ciencias Básicas, Facultad de Ciencias, Universidad del Bío Bío, Chillán, Chile; ^7^Laboratorio de Genómica, Centro de Biotecnología de los Recursos Naturales, Facultad de Ciencias Agrarias y Forestales, Universidad Católica del Maule, Talca, Chile; ^8^Departamento de Biotecnología, Facultad de Ciencias del Mar y Recursos Biológicos, Universidad de Antofagasta, Antofagasta, Chile; ^9^Departamento de Ciencias y Geografía, Facultad de Ciencias Naturales y Exactas y HUB Ambiental UPLA, Universidad de Playa Ancha, Valparaíso, Chile; ^10^Centro COPAS Coastal, Universidad de Concepción, Concepción, Chile; ^11^Laboratorio de Virología, Centro de Neurobiología y Fisiopatología Integrativa, Instituto de Química y Bioquímica, Facultad de Ciencias, Universidad de Valparaíso, Valparaíso, Chile; ^12^Center for Environmental Diagnostics and Bioremediation, University of West Florida, Pensacola, FL, United States; ^13^Departamento de Ciencias Farmacéuticas, Facultad de Ciencias, Universidad Catolica Del Norte, Antofagasta, Chile

**Keywords:** bioprospection, secondary metabolites, thermophilic bacteria, antimicrobial activity, bioactive compounds

## Abstract

Thermophilic microorganisms possess several adaptations to thrive in high temperature, which is reflected as biosynthesis of proteins and thermostable molecules, isolation and culture represent a great methodological challenge, therefore High throughput sequencing enables screening of the whole bacterial genome for functional potential, providing rapid and cost-effective information to guide targeted cultures for the identification and characterization of novel natural products. In this study, we isolated two thermophilic bacterial strains corresponding to *Bacillus* LB7 and *Streptomyces* LB8, from the microbial mats in the Atacama Desert. By combining genome mining, targeted cultures and biochemical characterization, we aimed to identify their capacity to synthesize bioactive compounds with antimicrobial properties. Additionally, we determined the capability to produce bioactive compounds under controlled *in vitro* assays and detected by determining their masses by Thin-Layer Chromatography/Mass Spectrometry (TLC/MS). Overall, both isolates can produce antimicrobial (e.g., Myxalamide C by-product) and antioxidants (e.g. Dihydroxymandelic Acid, Amide biotine and Flavone by-products) compounds. *Bacillus* LB7 strain possesses a more diverse repertoire with 51.95% of total metabolites unmatched, while *Streptomyces* LB8 favors mainly antioxidants, but has over 70% of unclassified compounds, highlighting the necessity to study and elucidate the structure of novel compounds. Based on these results, we postulate that the uncultured or rare cultured thermophiles inhabiting high-altitude hydrothermal ecosystems in the Atacama Desert offer a promising opportunity to the study of novel microbial bioactive compounds.

## Introduction

Microbial secondary metabolites are one of the most important sources of new compounds for drug discovery and development (Harvey et al., 2015; Tedesco et al., 2021). Harnessing bacteria for bioprospecting offers distinct advantages including their amenability to large-scale fermentation and genetic engineering to enhance metabolite production (Tedesco et al., 2021). Traditionally, bioactive compounds discovery has relied heavily on cultivating organisms in laboratory-controlled conditions; however, the vast majority of environmental taxa cannot be cultured using conventional methods. Nevertheless, the integration of high throughput sequencing and genome mining has revolutionized the field, providing powerful techniques to access the microbial biosynthetic potential enabling a targeted search for new bioactive secondary metabolites (Ziemert et al., 2016).

Current investigations have increasingly directed their focus toward extreme environments for having competitive conditions that promote the production of novel secondary metabolites as a defense or/and signaling strategy (Machado et al., 2015). Also, the enzymes and products formed under these challenging conditions are more physically and chemically stable when facing high pressure and temperature (Mashakhetri et al., 2024; Barzkar et al., 2024). These metabolites encompass a diverse array of compounds, including antimicrobial, antifungal, antitumor, antivirals, and antioxidants critical in modern medicine (Monciardini et al., 2014; Eltokhy et al., 2024; Palacios-Rodriguez et al., 2024).

The Andes highlands comprises an area with a mean altitude of 3,700 meters above sea level and presents several environmental conditions that push the boundaries of life, including high solar radiation, extreme changes in temperature and pH values, and high concentrations of metal (oids), among many others (Aguilar et al., 2016; Orellana et al., 2018). Thus, providing a natural laboratory to study poly-extremophile microorganisms and explore the production of diverse and novel bioactive compounds derived from them. One particular site of interest is the Lirima hydrothermal system, consisting of several ponds located in the Tarapacá Region in northern Chile (19.85^°^S, 68.91^°^W) southwest of the volcanic complex Aroma-Quimsachata that elevates up to 4,200–4,500 m asl, it is very remote and hard to access therefore is harbors great novelty potential (Tassi et al., 2010). The geological activity in the area generates optimal conditions for the development of poly-extremophile microorganisms with remarkable, yet understudied, metabolic potential (Filippidou et al., 2019; Pérez et al., 2018).

Previous investigations of poly-extremophile bacteria in the Andes highlands have reported promising candidates for biotechnological prospecting, especially *Streptomyces* that remains one of the richest sources for novel natural secondary metabolites (Donald et al., 2022) where environmental conditions trigger the production of diverse natural bioactive compounds (Przybylska-Balcerek et al., 2019). In particular, *Streptomyces leeuwenhoekii*, isolated from the Atacama Desert, was found to produce new bioactive compounds: chaxamycins and chaxalactins, with great antimicrobial properties (Elsayed et al., 2015; Rateb et al., 2018) and potent thermo-stable biocatalysts such as Baeyer–Villiger monooxygenases (Gran-Scheuch et al., 2018).

Additionally, two novel lasso-peptides with antitumoral properties were found to be produced by *Streptomyces* strains isolated from the hypersaline environment, the Chaxa lagoon in the Atacama Salar (Elsayed et al., 2015). A bacterial strain classified as *Streptomyces* isolated from Salar de Tara produced abenquines A–D with antimicrobial activity (Schulze-Makuch et al., 2018). Notably, recent genome mining efforts led to the discovery of a new antimicrobial class II lasso peptide (huascopeptin 1) from extremophile *Streptomyces* (Cortés-Albayay et al., 2019) among other compounds summarized in (Khan et al., 2021; Rateb et al., 2018).

Considering this revealing previous research in the Atacama Desert, the characterization of thermophilic bacteria isolated from poly-extreme environments remains a challenging task and the exploration of novel microbial compounds from high altitude hydrothermal systems in this region has remained limited. In this study we analyzed two thermophilic bacterial strains isolated from the Lirima hydrothermal system and investigated their potential to produce novel secondary metabolites with biotechnological significance, especially since they are classified in the *Streptomyces* and *Bacillus* genera, both groups are historically relevant in the biotechnological field. To achieve this, a combination of genomic mining, laboratory controlled microbiological assays and analytical tools were used. We hypothesize that the unique combination of environmental stresses, such as elevated temperature and ultraviolet radiation, have promoted the evolution of genetic survival mechanisms that favor fitness in *Bacillus* sp. LB7 and *Streptomyces* sp. LB8, some of them evidenced as the production of bioactive compounds, in particular antimicrobial and antioxidant molecules, in controlled laboratory conditions.

## Methods

### Study site and sampling

The Lirima hydrothermal system is located in the northern region of Chile over 4,000 m asl, it is difficult to access and is isolated from anthropological pressures (Supplementary Figure S1). This ecosystem comprises several environmental extreme parameters including high solar radiation (>1.120 Wm^−2^), high water temperatures (42–80°C) and low oxygen levels in water (Garreaud et al., 2003).

In November 2015, triplicate mat samples were collected for bacterial isolation from a hot spring (referred as P42 in Pérez et al., 2020) in the Lirima hydrothermal system (19.85°S; 68.91°W), samples consisted of a thick orange microbial covering most of the pond. P42 is a pond with 40 cm of depth and water temperature ranging from 42 to 53°C, that is colonized by a thick, compact, and orange microbial mat. Mat samples were collected in sterile 50 mL propylene tubes (Falcon™) and stored at 4°C upon collection until cultured in the laboratory. Physical and chemical parameters were obtained *in situ* with a multiparameter instrument (Hanna 9,829), including temperature, pH, conductivity, turbidity, and redox potential (Supplementary Table S1).

### Isolation of thermophilic bacteria from microbial mats

To select and isolate thermophilic bacteria, 1 g of microbial mat was transferred to a 50 mL sterile propylene tube (Falcon™) containing 30 mL of sterile Ringer solution (sodium chloride 0.85 g**·**L^−1^, potassium chloride 0.04 g**·**L^−1^, calcium chloride 0.034 g**·**L^−1^, pH 6.5). The tube was then heat-treated in successive rounds at 60°C, 70°C, 80°C and 90°C for 10 min each, plus an untreated control, followed by a 10-day incubation in dark conditions without agitation at 48°C (in triplicate). Then, 120 μL of these cultures were spread on plates with Difco™ Marine broth 2216 (MB) supplemented with 2% agar and 1% Dimethyl Sulfoxide (DMSO) and incubated for 3 days at 48°C inside a hand-made humidity chamber to maintain moist conditions under dark conditions.

Once isolated colonies were observed, they were transferred separately to plates containing Difco™ Marine Broth 2,216 media (supplemented with 2% w/v agar and 1% v/v DMSO). Two different isolates were selected according to colony color and morphology and transferred to sterile tubes containing Marine Broth supplemented with 1% v/v DMSO for axenic cultures for further experiments. Growth curves were carried out in different culture conditions in order to detect secondary metabolites biosynthesis (Supplementary Figure S2). For this, 96 well plates (Corning, Falcon) were inoculated with axenic cultures grown for 24 h at 45°C and adjusted to a OD_600_ nm of 0.01, and 150 μL of culture media (according to the conditions tested as listed below). The OD_600_ nm of the cultures were measured every 2 h for 56 h in a multiplate reader (Multiscan GO, Thermo Scientific). Additionally, for the growth curves at 48 and 55°C, each strain was grown in MB and minimal mineral medium (M9) broth and plated in media supplemented with 1% w/v agar, finally, after 24 h the Colony Forming Units were counted.

### Molecular identification of bacterial isolates

Taxonomic classification of isolated strains was carried out by 16S rRNA amplicon sequencing. Total DNA was extracted from 1 mL of axenic cultures using the Power Soil DNA (MoBio, Laboratories. Inc) following the manufacturer’s instructions. The 16S rRNA gene was then amplified using the Bacteria universal 27F and 1492R primers (Stackebrandt et al., 1993). PCR reactions contained 12.5 μL master mix buffer with SaphireAmp (2x), 5 μL of each nucleotide (10 μM), 2 μL of Bovine Serum Albumin (10 mg mL^−1^), 1 μL of forward and reverse primers and 2 μL of DNA template. Thermal cycling specifications were as follows: 94°C for 5 min, and 30 cycles of 94°C for 30 s, 55°C for 45 s, 72°C for 1 min, and a final extension of 72°C for 5 min (Stackebrandt et al., 1993). The presence of a 1,500 bp band was confirmed using 1% w/v agarose gels. The purified PCR products were then analyzed using the Sanger method (Macrogen Inc., Korea). For classification, the sequences were assembled using SPAdes v3.7 (Bankevich et al., 2012) using default settings and queried against GenBank (Benson et al., 2013) and SILVA v132 (Quast et al., 2012) 16S rRNA databases.

### Whole genome sequencing

One mL of axenic cultures of each selected strain was subjected to a thermal stress protocol (3 cycles of incubations at −80°C for 20 min and then 100°C for 10 min) to break the cell walls. Total DNA was extracted from those pretreated samples using the Power Soil DNA (MoBio, Labs) according to the manufacturer’s instructions. DNA integrity, quality, and quantity were verified using 1% w/v agarose gel electrophoresis and the Qubit dsDNA HS assay kit in the Qubit 2.0 fluorometer (Thermo Fisher Scientific, MA, United States). Later, DNA samples were submitted to the Microbial Genome Sequencing Center (MiGS; Pittsburgh, PA, United States) for paired end (2 × 151 bp) library construction using the Nextera DNA Library Prep kit and sequencing on an Illumina NextSeq 2000 platform.

### Genome mining for screening secondary metabolite potential

The quality of raw sequencing data was evaluated with FastQC v0.11.8 (Andrews, 2010) and filtering/trimming (Ns = 0, read length ≥ 100 bp and Q ≥ 30) using PRINSEQ v0.20.4 (Schmieder and Edwards, 2011). Pre-processed reads were *de novo* assembled with SPAdes v3.7 (Bankevich et al., 2012). Genome assemblies were evaluated by statistical values calculation with QUAST v5.0.2 (Gurevich et al., 2013) and completeness analysis through the search of bacterial orthologous genes (OrthoDB v9 database: Zdobnov et al., 2017), using BUSCO v3 (Waterhouse et al., 2017). The resulting contigs were annotated with Prokka v1.13.3 (Seemann, 2014), and eggNOG-mapper v1.0.3 (Huerta-Cepas et al., 2017). Identification of potential regions associated with secondary metabolites production were performed using the platform antiSMASH (Medema et al., 2011). The whole genome for both strains was deposited at DDBJ/ENA/GenBank under Bioproject number PRJNA956856 (Biosamples: SAMN34378102 and SAMN34231586).

### Characterization of secondary metabolites and the conditions that promote their production

The initial condition consists of *Bacillus* LB7 and *Streptomyces* LB8 bacterial strains grown in M9 media at 48°C with constant agitation of 80 rpm in all instances unless stated otherwise. The following culture conditions were assayed to produce secondary metabolites, aiming to trigger the activation of bioactive compounds with challenging conditions including variations in nutrient composition, temperature, salinity, and pH values (Table 1).

**Table 1:** Treatments used for secondary compounds evaluation.

Parameter	Treatments		
	T1	T2	T3
pH (adjusted with sodium hydroxide 3 M or 10% w v^−1^ hydrochloric acid)	4	8	12
NaCl (M)	0.6	1.5	3
Glucose (M)	0.01	0.005	
Temperature (°C)	37	58	
Nutrient (media)	Difco Marine broth 2,216 + DMSO	M9 (M9, Casamino acid 1 g**·**L^−1^, disodium phosphate 6 g**·**L^−1^, potassium phosphate 3 g**·**L^−1^, ammonium chloride 1 g**·**L^−1^, sodium chloride 21 g**·**L^−1^)	
UV	Darkness	UVC pulses	

The UV pulses were carried in axenic cultures maintained at 48°C in M9 media and incubated in darkness or treated with germicide UVC pulses (100–280 nm, 40 μW/cm^2^). For these incubations, sterile transparent Whirlpack bags (Aas et al., 1996) were used with 80 mL of M9 media, inoculated with a single colony, and incubated for 48 h. Cultures were then either maintained in darkness or treated with radiation. Treatments in darkness were maintained with constant agitation of 80 rpm at 48°C, while the cultures for UV treatment were exposed to UVC during 5, 10 and 20 min in independent bags, using a UVC lamp ESCO CRF-UV 15A. After exposure, the cultures were incubated with constant agitation of 80 rpm at 48°C for 30 min for recovery, and then were exposed to a second UVC pulse for the time ranges previously mentioned. All experiments were performed in three replicates.

### Obtaining organic extracts from the bacterial cultures

Bacterial culture (500 mL) under each treatment condition was extracted with ethyl acetate (1,1 v v^−1^). The aqueous phase was discarded, and the organic phase was mixed with HPLC grade water in a 1:2 (v v^−1^) ratio. Extracts were concentrated to dryness at 60°C on Quickfit RE100 rotary evaporator. The remaining moisture was removed by bubbling with nitrogen gas, and re-dissolved in ethanol:water (4:1 (v v^−1^)) solution (500 μL), for usage in the following experiments.

### Chromatography

Extracts were screened for bioactive compounds with antimicrobial activity by High Performance Thin-Layer Chromatography (HPTLC). The samples were applied onto 20 × 10 cm HPTLC plates silica gel 60 F_254_ using an CAMAG (Muttenz, Switzerland) automatic Thin Layer Chromatography (TLC) sampler (ATS4) set as follows: band length 8 mm, track distance 10 mm, dosage speed 40–150 nL s^−1^ and first application x-axis and y-axis at 10 mm, using the corresponding media as control. Chromatography was performed in a CAMAG 20 × 10 cm twin-trough chamber up to a migration distance of 80 mm using a mobile phase composed of toluene: ethyl acetate (3.15:1.85 v/v). After drying for 10 min at 70°C on CAMAG TLC plate heater, plate images were photo-documented under 254 and 366 nm light illumination (reflectance) using a CAMAG Reprostar 3 documentation system. All the instruments were controlled through CAMAG WinCats 1.4.7 software. Unless stated otherwise, extracts were applied in triplicate dividing the HPTLC plate in two sections: the first section was used for bioassay, the second for Mass Spectrometry analysis.

### Antibacterial activity bioassay

Bioautography in planar chromatography was used to determine the antimicrobial activity in the organic extracts from *Bacillus* LB7 and *Streptomyces* LB8 following the method described by (Jamshidi-Aidji and Morlock, 2015) with slight modifications. Briefly, plates were automatically immersed (3.5 cm s^−1^) during 6 s into bacterial culture (0.035 ≤ OD_600_ ≤ 0.1) by means of CAMAG immersion device. Then, the plate was incubated horizontally at 37°C for 2 h, under aerobic conditions, over a horizontal stand inside a closed moisture chamber. After incubation, the plate was then sprayed 0.2% w v^−1^ phosphate-buffered saline thiazolyl blue tetrazolium bromide (MTT) staining solution (pH 7.4) with an automatic Merck TLC-sprayed and incubated at 37°C for 30 min. The plate was then dried on a TLC plate heater for 5 min at 45°C. Plate image was photo-documented under white light illumination (reflectance) using CAMAG Reprostar 3 documentation system. Metabolically active microorganisms reduce MTT to formazan purple (associated with the oxidation of cellular oxidoreductase enzymes dependent on NADPH), while colorless zones on this purple background indicate the presence of antibacterial compounds (Berridge et al., 2005).

### Mass spectrometry detection of antibacterial compounds

Bioactive bands were directly eluted through the Thin-Layer Chromatography/Mass Spectrometry (TLC/MS) interface (oval elution head, 4×2 mm) to electrospray ionization (ESI) source of Shimadzu (Kyoto, Japan) LCMS 8030 triple quadrupole mass spectrometer (Aranda and Morlock, 2007). Methanol was used as an elution solvent at a flow rate of 0.1 mL min^−1^ controlled by the Shimadzu HPLC pump. MS analysis was carried out with the following parameters: ESI (+) voltage 3.5 kV, nebulizer gas flow (N_2_) 3 L min^−1^, drying gas flow (N_2_) 15 L min^−1^, desolvation line (DL) temperature 250°C and heat block temperature 400°C. Identification was carried out using the second plate section (without bioassay) by eluting the band directly to the ESI interface of the MS using the CAMAG TLC-MS interface. Data was acquired in full scan mode (*m/z* 100–2000) and analyzed by Shimadzu LabSolution 5.51 software. Finally, the mass corresponding to each peak was recorded and compared to the “Metabolic *in Silico* Network Expansions” (MINEs) database to determine the chemical nature of the compounds as described by (Galarce-Bustos et al., 2019).

## Results

Two colored bacterial isolates were obtained after heat-shock, one orange/reddish and one black as previously described in the Methods section. The strain LB7 grew as reddish colonies and its 16S rRNA sequence had 100% similarity to *Bacillus haynesii* and the LB8 strain formed black colonies and had 99.92% similarity to *Streptomyces thermogriseus* using NCBI Blast database. These two strains were selected for further characterization among the dozens of other isolates given their colony morphology and coloration and their preliminary identification by 16S sequence similarity belonging to two genera that are very relevant for biotechnology and pharmaceutical applications.

**Figure 1:**
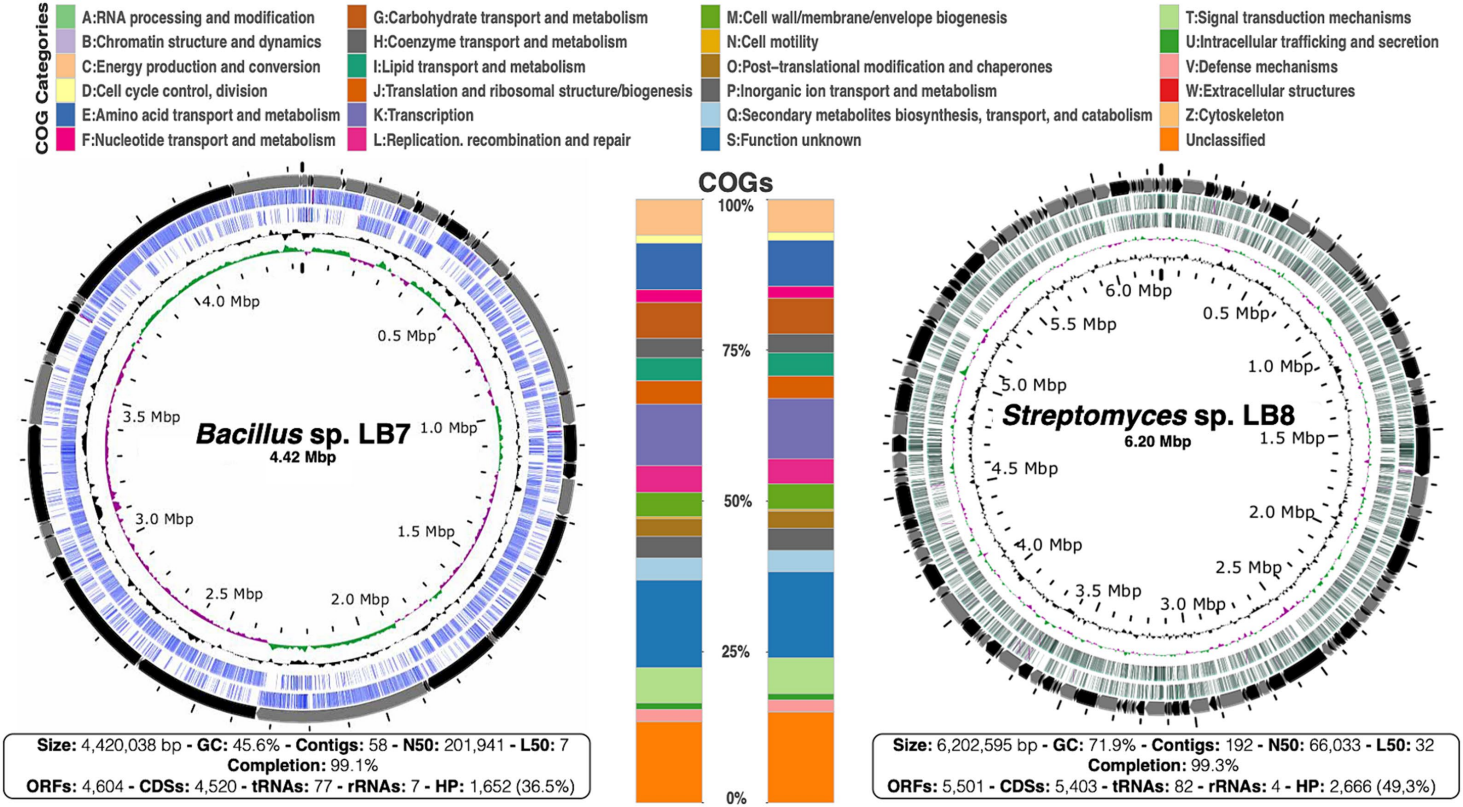
Genomes of *Bacillus* sp. LB7 and *Streptomyces* sp. LB8. Circular map of both draft genomes, concentric circles represent (from outside to inside): contigs (alternately colored black and gray), ORFs (open reading frames) in both strands (colored in blue for LB7 and in green for LB8), GC content (in black) and GC skew (+ in green and −in pink). The stacked bars at the center correspond to the composition in both genomes according to COG categories; each predicted protein was functionally classified (in color-coded categories). And the bottom squares show the genome assembly characteristics and attributes for each genome.

Analyses of the assembled genomes revealed genome sizes of approximately 4 and 6 Kb for the LB7 and LB8 strains, respectively. The sequencing depth was 133x for LB7 and 108x for LB8, further sequencing details are shown in Figure 1. The genomes have a large proportion of unclassified proteins (36.5 and 49.3% for *Bacillus* sp. LB7 and *Streptomyces* sp. LB8, respectively), representing genomic attributes that have not been described and May represent great potential for the discovery of novel bioactive compounds.

The most enriched metabolic functions in the genomes from strains LB7 and LB8 are transcription, transport, as well as carbohydrates and amino acids, maintaining basal functions and cell viability. Also, our aim is to characterize the potential of these strains for secondary metabolite biosynthesis, signal transduction, and ribosomal structure/biogenesis (Figure 1) that are also well represented in both genomes.

### Genetic potential to produce secondary metabolites of the *Bacillus* LB7 and *Streptomyces* LB8 strains

A genome-wide analysis shows that both isolates have several gene clusters associated with the production of a variety of secondary metabolites. For instance, the genome of the LB7 strain possesses eleven regions with genetic functions related to the production of secondary metabolites, from which five were classified as antibiotic, antifungal, surfactant, and catechol-based siderophore (Table 2).

**Table 2:** Genetic clusters associated with secondary metabolite production on the *Bacillus* LB7 strain genome.

Region	Type	Nucleotide		Most similar known cluster	Similarity	Associated function
		From	To			
1	RiPP-like	4,945	14,996	–	–	–
2	NRPS	110,191	191,630	Bacitracin	100%	Antibiotic
3	T3PKS	321,196	362,293	–	–	–
4	NRPS, T1PKS, terpene	379,128	456,657	–	–	–
5	NRPS	1,782,655	1,836,429	Lichenysin	100%	Antibiotic, Surfactant
6	NRPS, betalactone	2,283,464	2,333,014	Fengycin	86%	Antibiotic, antifungal
7	Siderophore	2,467,109	2,482,499	–	–	–
8	CDPS	2,831,750	2,852,499	–	–	–
9	NRPS	3,334,166	3,381,323	Bacillibactin	53%	Antibiotic, antitumor, siderophores
10	Lassopeptide	3,488,454	3,510,927	–	–	–
11	Thiopeptide, RiPP-like	3,734,656	3,775,831	Butirosin A/Butirosin B	7%	Aminoglycoside antibiotic

Clusters unmatched with known clusters are denoted with “-.”

The genome of the LB8 strain showed twenty-three regions with secondary metabolite potential, from which seventeen were classified as clusters that codify for peptide antibacterial, antifungal, lasso peptides, stress response metabolites, phytotoxic, antitumor, antioxidant, pigments, antiviral and immunosuppressant functions (Table 3). Over 25% of the detected regions were associated with secondary metabolites based on their structure but remained as unclassified as they do not match any known clusters on the MIBiG repository (Terlouw et al., 2023).

**Table 3:** Genetic clusters associated with secondary metabolite production on the *Streptomyces* LB8 strain genome.

Region		Nucleotide				
	Type	From	To	Most similar known cluster	Similarity	Associated function
1	Redox-cofactor	50,214	73,001	–	–	–
2	NAPAA	480,324	514,744	Stenothricin	13%	Antibiotic
3	Thiopeptide, LAP, siderophore	1,569,742	1,605,016	–	–	–
4	Lassopeptide	1,807,832	1,830,430	Chaxapeptin	28%	Lassopeptides
5	Siderophore	2,000,875	2,011,432	-	-	-
6	Ectoine	2,108,211	2,118,615	Ectoine	100%	Osmotic stress reaction
7	RiPP-like	2,442,726	2,452,532	–	–	–
8	T3PKS	2,458,946	2,500,004	Herboxidiene	5%	Antibacterial, antitumor
9	Terpene	3,025,566	3,049,031	Carotenoid	63%	Antibiotic (pigment)
10	Butyrolactone	3,558,699	3,567,982	Chondrochloren	50%	Antibiotic (latam among others), antitumor, antifungal
11	T2PKS	3,343,401	3,413,785	Mayamycin	72%	Antibiotic (macrolides), antitumor
12	Butyrolactone	3,558,699	3,567,892	Neocarzinostatin	6%	Antitumor antibiotic
13	Betalactone, siderophore, lanthipeptide-class-i	3,659,619	3,713,166	Desferrioxamine	83%	Antifungal, siderophores
14	NRPS-like, butyrolactone	3,714,886	3,756,801	Lactonamycin	5%	Antibacterial
15	Lanthipeptide-class-I	4,176,064	4,201,341	–	–	–
16	PKS-like, T1PKS	4,322,763	4,370,474	Arsono-polyketide	75%	Polyketide
17	NRPS, NAPAA, terpene	4,773,300	4,816,581	Stenothricin	13%	Antibiotic (including lipopeptides)
18	T2PKS	4,960,865	5,033,380	Spore pigment	83%	Antibiotic, antitumor, antifungal
19	RiPP-like	5,835,922	5,884,571	–	–	–
20	Butyrolactone	5,472,550	5,483,563	Cyphomycin	6%	Antifungal, antiplasmodial
21	PKS-like	5,835,922	5,884,571	Sanglifehrin	6%	Antibiotic, antitumor, immunosuppression
22	Terpene	5,889,153	5,909,308	Albaflavenone	100%	Antibacterial
23	Terpene	5,925,036	5,951,725	Hopene	53%	Antimicrobial and Antitumor

Clusters unmatched with known clusters are denoted with “-.”

### Biocompounds produced by *Bacillus* BL7 and *Streptomyces* LB8

Thin-Layer Chromatography/Mass Spectrometry (TLC/MS) enables separation and identification of the secondary metabolites contained in each organic extract, allowing to selectively determine each mass and by comparing to existing databases identify the most similar compound (Feliatra et al., 2021; Mukherjee et al., 2024). The metabolites profiles produced under each assayed condition indicate that the strains have differential response for generating secondary metabolites. The identification of the biocompound secreted in response to each treatment are listed in Tables 3, 4.

**Table 4:** Identification of antioxidant and antimicrobial compounds obtained from *Bacillus* LB7 by the mass spectrometer analysis, based on the Metabolic *in silico* Network Expansions database.

Strain	Band	m/z	Identification (MINE)	MINE code	Nature	Associated function
LB7 grown with 0.01 M Glucose	A	232.05	Phosphinate by-product	566,860	–	Antifungal, herbicidal, antiparasitic, antimicrobial
	B	275.2	Myxalamide C by-product	141,593	–	Antibacterial
LB7 grown at pH 12	A	265	Dihydroxymandelic Acid by-product	94,678	Phenol	Antioxidant-metabolite of norepinephrine
LB7 grown with 1,5 M NaCl	A	226.1	Amide biotine by-product	261,939	Carboxamide	Antioxidant, antibacterial
	A	232.05	Phosphinate by-product	566,860	Acid Anhydride acyclic phosphorus	Antifungal, herbicidal, antiparasitic, antimicrobial
	A	240.1	Flavone by-product	231,654	Flavonoid	Antioxidant, anti-inflammatory, anti-mutagenesis
	A	232.05	Phosphinate by-product	566,860	Acid Anhydride acyclic phosphorus	Antifungal, herbicidal, antiparasitic, antimicrobial
LB7 grown at 37°C	A	240.1	Flavone by-product	231,654	Flavonoid	Antioxidant, anti-inflammatory anti-mutagenesis

Each strain has a different profile of identified metabolites, including antioxidants such as 15,9′-dicis-phytofluene by-product and antimicrobials including Aurachin by-product and Neomethymicin or 10-Deoxymetinolide by product (Table 5). The LB8 strain produces siderophores and molecules associated with radiation response, critical for survival in their natural environment in a high-altitude hydrothermal system in the Altiplano (Supplementary Table S3). MINE code corresponds to the KEGG codification.

**Table 5:** Identification of antioxidant and antimicrobial compounds obtained from *Streptomyces* LB8 by the mass spectrometer analysis, based on the metabolic *in silico* network expansions database.

Strain	Band	m/z	Identification (MINE)	MINE code	Nature	Associated function
LB8 grown at 58°C	B	355.3	15,9′-dicis-phytofluene by-product	255,133	Isoprenoids	Carotenoid production, antioxidant
LB8 grown with 0.005 M Glucose	A	362.25	Aurachin by-product	337,935	Alkaloid	Antibacterial
	B	313.2	Neomethymicin or 10- Deoxymetinolide by product	158,429	PKS (Macrolides and Lactone)	Antibacterial
	C	312.2	Neomethymicin or 10- Deoxymetinolide by-product	158,429	PKS (Macrolides and Lactone)	Antibacterial

MINE code corresponds to the KEGG codification.

The chromatogram of the LB7 strain showed a higher number of bands when the isolate was grown in media supplemented with NaCl 1.5 M, suggesting that higher salt concentration promoted secondary metabolite production. In contrast, chromatograms obtained from the LB8 strain showed more bands when cultures were grown in media supplemented with glucose 0.005 M and using Marine broth (0.5 M NaCl); suggesting that for this *Streptomyces* strain, the nutrient availability is directly associated with the production of secondary metabolites (Supplementary Figure S3A).

Bioautobiography assay shows that the LB7 strain produces secondary metabolites with antimicrobial activity when cultures were grown in (i) Marine broth (1 band); (ii) at 37°C (2 bands); (iii) at pH 12 (2 bands); and (iv) with glucose 1 mM. The strain LB8 produces compounds with antimicrobial activity when the bacteria were grown (i) in Marine Broth (1 band) and (ii) at 58°C (1 band) (Supplementary Figure S3B).

**Figure 2:**
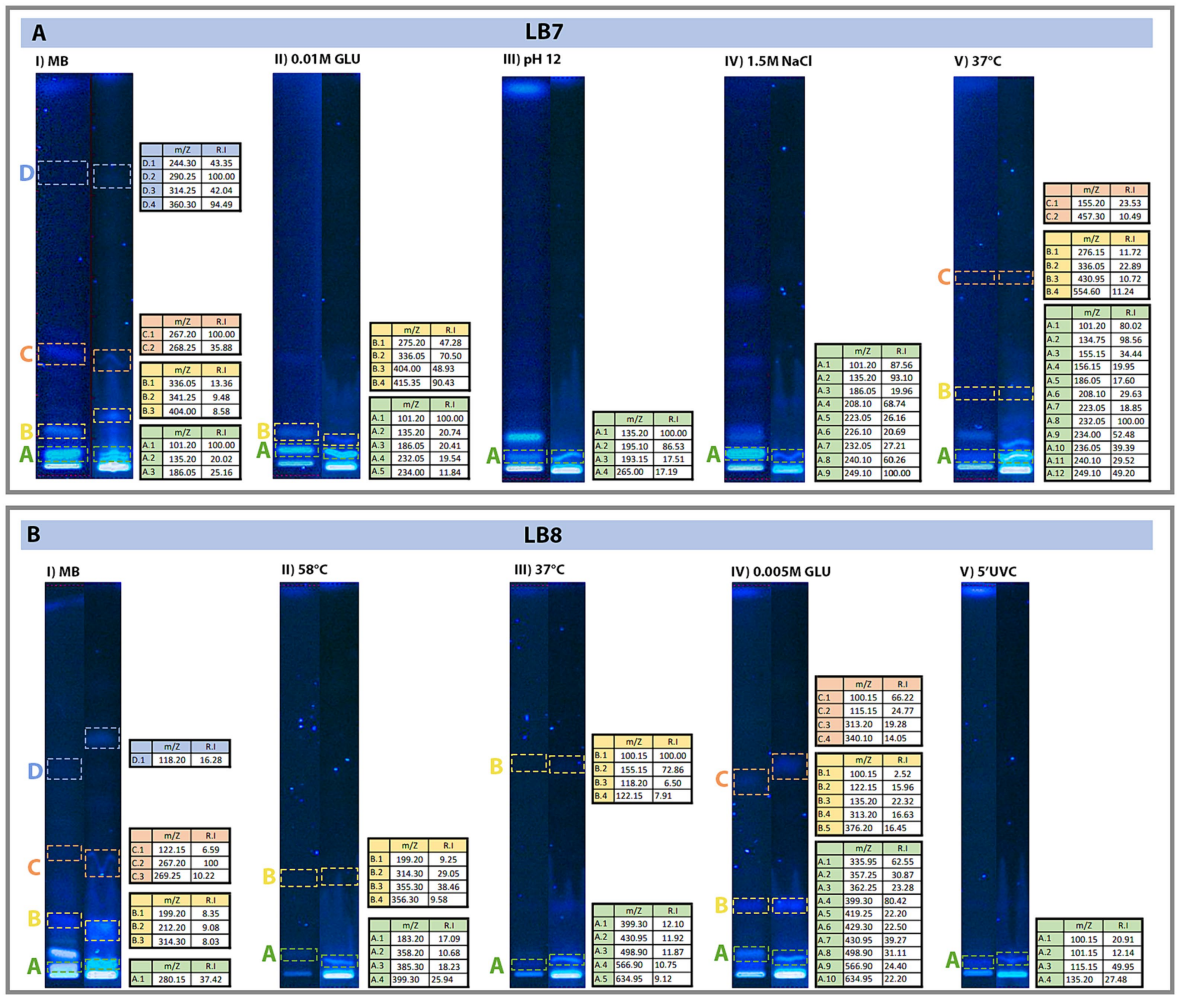
Chromatograms showing the selected bands from the bioactive compounds for the mass spectrometry analysis showing the original HTLC plate (left) and the HTLC used for mass spectrometry (right). Displaying **(A)** Extracts from the LB7 strain. **(B)** Extracts from the LB8 strain. Bands represent the migration of the different metabolites. Color squares highlight the used bands, and Table on the right panel shows concentration of each sample.

The HTLC results indicate that there are up to four distinct bands, in order to identify the compounds corresponding to each band we carried out MS/MS analysis. While some compounds were identified, the majority remains uncharacterized (LB7: 51.95% and LB8: 70.83%), representing a rich source of potential novel bioactive compounds to be explored in detail in future research (Figure 2).

Among the chemical compounds identified through mass spectrometry are those commonly associated with antioxidant activity such as Amide biotine and Dihydroxymandelic Acid by-products (Table 4). Also, the bacteria secrete compounds associated with antimicrobial activity, such Flavone, Phosphinate, and Myxalamide C by-products (Table 4) among other functions (Supplementary Table S2). Band A showed the highest peak in most conditions evaluated (Supplementary Figure S4) corresponding to antioxidants, chelating, anti-inflammatory, cytotoxic, antimicrobial and some with unknown activities.

The combination of classical microbiology approaches and combinations of heat treatments used to isolate extremophile bacteria enabled to isolate two representatives of the *Streptomyces* and *Bacillus* genera, moreover genomic bioprospecting indicated that these strains had the potential to produce several compounds of biotechnological interest. Finally, by using thin layer chromatography coupled to mass spectrometry we were able to determine the nature of the secondary metabolites secreted in response to several conditions in laboratory-controlled experiments, highlighting the great potential that these strains have for pharmacological applications and large-scale production under industrial production of thermostable molecules produced by thermophiles inhabiting a very extreme and under studied environment.

## Discussion

Poly-extreme ecosystems represent natural laboratories for the bioprospection of novel or uncharacterized secondary metabolites with diverse biotechnological and pharmaceutical applications (Machado et al., 2015; Orellana et al., 2018; Mashakhetri et al., 2024). Albeit the isolation of culturable representatives obtained from such environments represent a challenge, as axenic culture is required for large-scale characterization and isolation of bioactive compounds. In this study we isolated two thermophilic bacterial strains from a microbial mat in a high-altitude hydrothermal system and explored the potential biosynthesis of antimicrobial, antioxidants and other secondary metabolites of biotechnological interest.

Given the current scenario of multi-resistant and novel pathogens proliferation, it is critical to explore novel compounds that can assist in the patient treatment and alleviate the associated strain on society (Sass, 2023; Eshboev et al., 2024). The results obtained here show that the genomes of the *Bacillus* LB7 and *Streptomyces* LB8 strains possess bacterial gene clusters for producing compounds with a plethora of relevant antimicrobial, such as polyketide synthase (PKS), non-ribosomal peptide synthase (NRPS), and post-translationally modified peptides (RiPP), as well as antifungal, biosurfactants, chelating agents, antitumoral, immunosuppressive, and antiviral compounds, which are critical attributes for the surviving in poly-extreme environments for defense from predators, obtaining overall fitness and competition for resources such as energy production (Kumar et al., 2022; Van Der Meij et al., 2017).

*Bacillus* LB7 and *Streptomyces* LB8 showed more than half of the detected coding regions were unclassified or unmatched in the MIBiG repository (Terlouw et al., 2023), suggesting that they are novel and uncharacterized bioactive compounds that are still challenging to identify. This finding has been observed in other extremophile surveys including microorganisms from glaciers, desert, deep-sea sediments (Sayed et al., 2020; Tedesco et al., 2021; Thompson and Gilmore, 2023; Xie and Pathom-Aree, 2021), highlighting the relevance of characterizing these unique microbial communities and bioprospecting for novel bioactive compounds. Secondary metabolite production pathways evolved to ensure microbial survival, and it requires a great amount of energy and resources to produce, suggesting a critical necessary biological function (Sekurova et al., 2019).

### Different stress pressures trigger the production of specific secondary metabolites with antimicrobial activity

Secondary metabolites production is part of the overall adaptation of extremophiles to thrive in oligotrophic and highly competitive ecosystems with challenging environmental conditions (Filippidou et al., 2019). Here we show using laboratory assays that some of the molecules were produced in response to different challenges, for example Mersacilic acid by product was secreted in response to temperature and nutrient stress. The results presented here indicate that the secondary metabolite response in both isolates is specific according to selective pressures. We determined a total of 21 potential bioactive compounds from the *Bacillus* LB7 and *Streptomyces* LB8 strains; however, around 50% of the bands obtained in this study did not match with any other known compounds, suggesting novel natural products that are yet to be characterized.

A higher concentration of NaCl (1.5 M) induced greater production of secondary metabolites compared to the patterns obtained when bacteria were grown in media supplemented with 0.6 M NaCl, suggesting increasing salinity triggers a response to survive the damage caused by changes in osmotic stress. To cope with high salt concentrations in the environments, microorganisms produce and accumulate osmoprotectants inside the cell even though they are energy costly these compounds provide a rapid and flexible mechanism to response to osmotic stress (Bremer and Krämer, 2019; Gunde-Cimerman et al., 2018; Oren, 1999). Some of the osmoregulants found in this study include isoprenoids (and derivatives like terpenes), betaine, glutamate, polyols (mannitol), amino acids (proline), among others to contribute to osmolality equilibrium; these compounds can also have antioxidant activity (Sévin et al., 2016). These kinds of molecules were identified in this study as produced by LB7 and LB8, particularly in response to challenges to NaCl and temperature.

Additionally, both studied strains had the capability to produce compounds with antimicrobial activity, as evidenced by the growth inhibition of *B. subtilis*. In this case, temperature plays a relevant role in activating mechanisms. When the LB7 strain was grown at 37°C, it produced at least two different molecules that inhibited the growth of *B. subtilis*. In comparison, when incubated at 58°C, the LB8 strain produced at least one molecule that inhibited this pathogen. Therefore, both strains were capable of producing antimicrobial compounds; although, the triggering mechanisms were activated at different temperatures.

The media used for cell culture also directly affected the antimicrobial compounds synthesized by the bacteria. The natural environment in Lirima is slightly oligotrophic (Pérez et al., 2017), thus elevated nutrient concentration might activate a defense response as the competition increases, also the presence of higher nutrient availability enables the production of more complex and diverse repertoire of compounds as found in cultures grown in Marine broth.

Both *Bacillus* and *Streptomyces* strains in this study produce an array of molecules in response to each tested challenge, some with specific functions associated to the environmental conditions in which these bacteria inhabit, for instance metal (oids) resistance, quorum sensing, and others (Albarracín et al., 2016; Tapia et al., 2018). Also, they produce a variety of scaffolds for a number of molecules, namely terpenes, octanes, phenols (Greenhagen and Chappell, 2001; Marson, 2011; Tahir et al., 2021). However, many of these biocompounds have functions of biotechnological interest, including pharmaceutical -antimicrobial, antioxidants, anticholinergic-and industrial-food preservatives, chelators- (Supplementary Table S2). Although some of these molecules can be chemically synthesized or obtained from other bacteria, we propose that given the current scenario of multidrug emergency and industrial eco-sustainability the novelty of the mechanism and inherit resistance of this thermophiles molecules can aid in looking for more energy-efficient procedures as well as deeper understanding for more stable pharmaceutical compounds (Chung et al., 2020; Gomes et al., 2016; Seale et al., 2015; Zeldes et al., 2015). Also, it is necessary to test for biological activity each compound separately to characterize their function, in this study we only tested for antibacterial activity.

The identification by MS showed that *Bacillus* LB7 grown with glucose 10 mM, produces Myxalamid C with *m/z* 275.2 (B.1), an antibiotic originally described in Myxobacteria, Gram-negative soil bacteria, and has antimicrobial activity against yeasts, molds and some Gram-positive bacteria (Silakowski et al., 2001). Also, when cultures were grown at 37°C, there were two bands with antimicrobial properties, one of which is associated with 2-phenyl-1-(1,2,4-triazol-1-yl)-3-trimethylsilyl propane-2-ol, with *m/z* 276.15 (B.1); while the other is another Thiazole by-product with *m/z* 236.05. Both compounds have a wide range of antimicrobial activity and have been previously reported as antivirals, antibacterials (Gram-positive and negative) as well as antifungal activities (Mohanty et al., 2022).

These types of molecules are scaffolds that can produce great variety of antimicrobials (Mishra et al., 2020; Petrou et al., 2021), that can even be more potent and effective than currently widely used antibiotics such as penicillin (Haroun et al., 2016). For example, 5-acetyl-4-methyl-2-(3-pyridyl) thiazole has proven activity against multiresistant bacteria *Staphylococcus aureus* (MRSA) and *Eggerthia catenaformis* (Ibrahim et al., 2021). Also, the production of antimicrobials by other *Bacillus* species have previously included the synthesis of fengycin, bacillomycin, macrolactin H, bacillaene, difficidin, siderophores, bacteriocin, among many others (e.g., Wang et al., 2024; Eltokhy et al., 2024) some of which were also detected in our genome mining approach (Table 4).

In contrast, the *Streptomyces* LB8 strain, when grown in media supplemented with glucose, produced by-products associated with the type II polyketide Aurachin (362.25 *m/z*, A.3) a widespread antimicrobial, antifungal and antiplasmodial agent that belongs to a family of natural products characterized by a quinoline chromophore substituted in position 3 or 4 by a farnesyl chain. This is a representative example of the results found in genome-mining, i.e., PKS, and the protein found in the chromatogram. This molecule was first identified as product of *Stigmatella aurantiaca*, however more recently it was also found to be produced by *Streptomyces venezuelae* (Kunze et al., 1987; Sandmann et al., 2007; Höfle and Irschik, 2008). In addition, Aurachins act as inhibitors of cytochrome complexes in the electron transport chain as they are structurally similar to ubiquinol and vitamin K (Debnath et al., 2012; Jünemann et al., 1997).

Among the bioactives compounds the results show that macrolides, such as 10-deoxymethynolide, converts it into a total of four compounds, including neomethymycin (Aldrich et al., 2005; Lambalot and Cane, 1992; Xuan et al., 2008; Zhang and Sherman, 2001). According to the determined mases (313,2 *m/z*, B.4) it could be Neomethymycin or 10-Deoxymethynolide, both macrolides that were previously identified in the repertoire of *Streptomyces venezuelae* and have strong antibiotic activity against Gram-negative and-positive, anaerobic and aerobic bacteria (Chen et al., 2001; Cunha et al., 1982; Graziani et al., 1998).

Overall, our results describe the biotechnology potential of two thermophilic bacteria belonging to *Bacillus* and *Streptomyces* genera, isolated from a high-altitude hydrothermal ecosystem. We found that the laboratory pressures assayed, including nutrient availability, pH values and temperature, favor a differential secondary metabolite production with great novelty and diversity. *Bacillus* LB7 produced greater diversity of compounds with more pharmacological applicable molecules including medication for neurological symptoms—e.g., procyclidine-, infections treatments, among others (Supplementary Table S2). However, *Streptomyces* LB8 holds more novelty as over 30 molecules are unmatched to the current databases, also this strain produced a greater number of hydrocarbon scaffolds and pigments allowing versatility to produce a wide array of biocompounds (radiation and antioxidant properties—e.g., Spiridine-, Supplementary Table S3).

There are over 50 secondary metabolites with unknown functions synthesized by *Bacillus* LB7 and *Streptomyces* LB8, which might potentially hold relevant compounds unknown to modern science. Both approaches used in this report, namely genome mining and thin layer chromatography, provide clues that there is functional potential in the genome of the bacteria as well as the production of compounds with experimentally validated antimicrobial activity, however the characterization of these unknown molecules remains as the main challenge. Poly-extreme environments such as the Lirima hydrothermal system, have taxa and metabolites novelty, whose particularity is that coming from a thermal source, the molecules must be more resistant to industrial processes that include thermal treatments (Ibrahim and Ma, 2017). Further studies that aim to purify and chemically characterize each compound will contribute in the future to the knowledge of bioactive compounds that can be obtained from underexplored thermophilic microorganisms. These findings contribute to the importance of bioprospecting novel drugs and natural products from thermophilic microorganisms inhabiting unexplored or minimally explored remote ecosystems.

## Conclusion

Bacterial strains belonging to the genera *Bacillus* and *Streptomyces* isolated from a high-altitude hydrothermal system in the Atacama Desert; possess the potential for the production of multiple secondary metabolites with bioactive properties of pharmaceutical interest, including compounds with antimicrobial, antioxidant, antitumoral properties, among many other functions to be explored.

## Data Availability

The datasets presented in this study can be found in online repositories. The Whole Genome Shotgun data for both strains has been deposited at DDBJ/ENA/GenBank under Bioproject PRJNA956856 (Biosamples: SAMN34378102 and SAMN34231586).
