# Supplementary figures and images for: Secondary metabolites with antimicrobial activity produced by thermophilic bacteria from a high-altitude hydrothermal system
Source: Front Microbiol. 2024 Sep 30;15:1477458. doi: 10.3389/fmicb.2024.1477458 (PMC11474921; doi:10.3389/fmicb.2024.1477458)

# PAMPA LIRIMA - TARAPACÁ REGION, CHILE

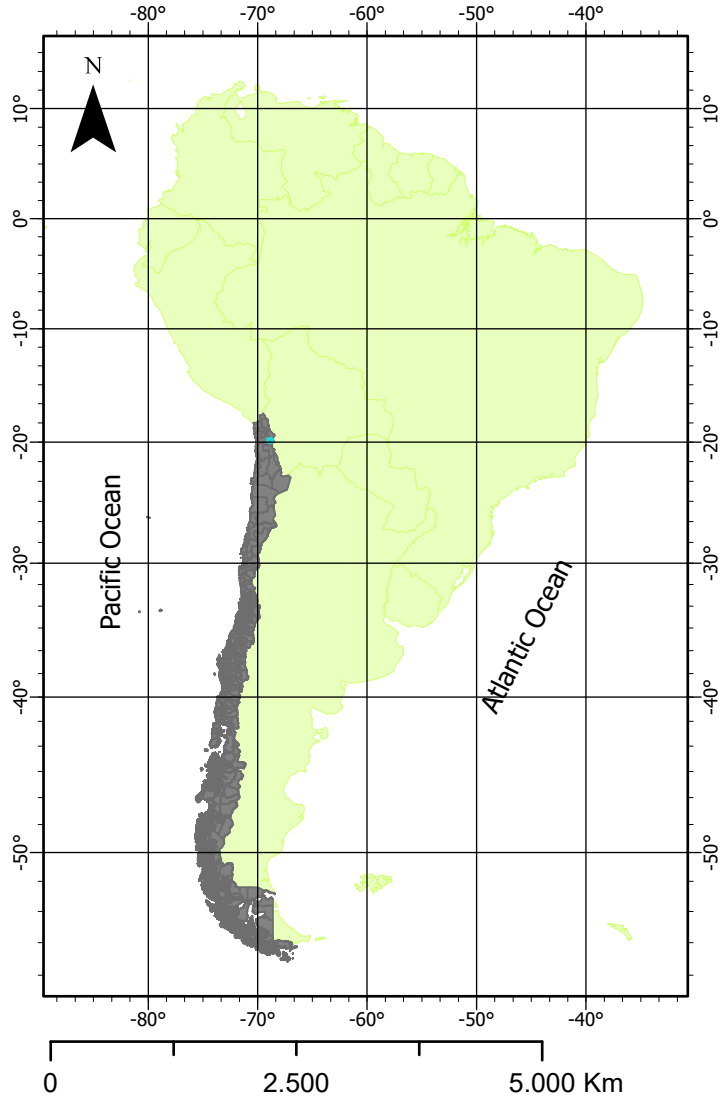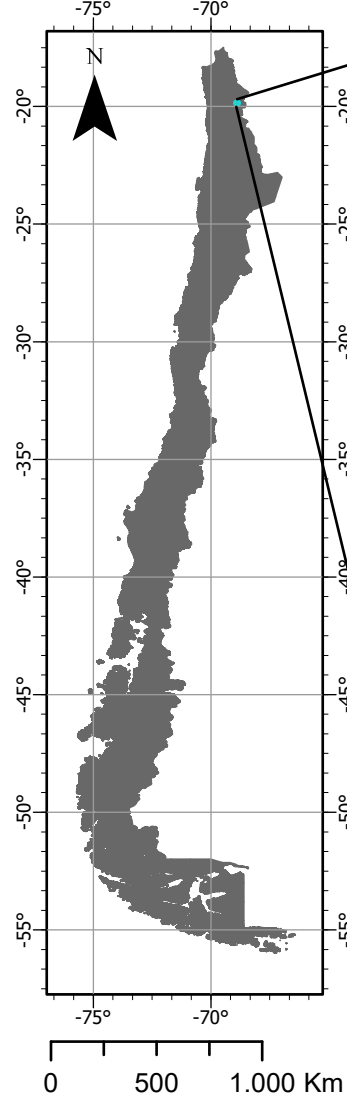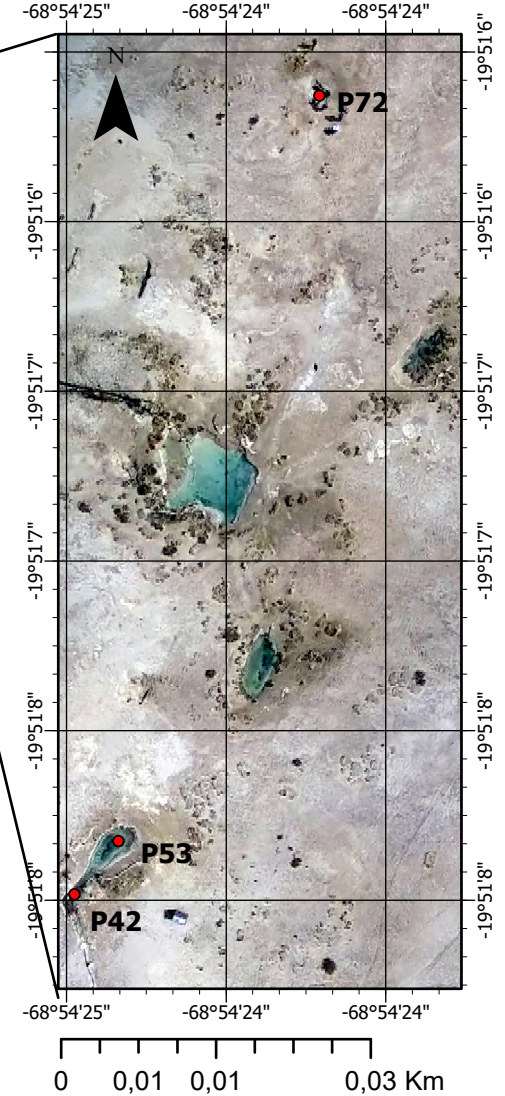

Supplement: Supplementary file 1 [file Data_Sheet_1.PDF]
