# Supplementary material for: Secondary metabolites with antimicrobial activity produced by thermophilic bacteria from a high-altitude hydrothermal system
Source: Front Microbiol. 2024 Sep 30;15:1477458. doi: 10.3389/fmicb.2024.1477458 (PMC11474921; doi:10.3389/fmicb.2024.1477458)
Supplement: Supplementary file 2 [file Data_Sheet_2.PDF]

A)

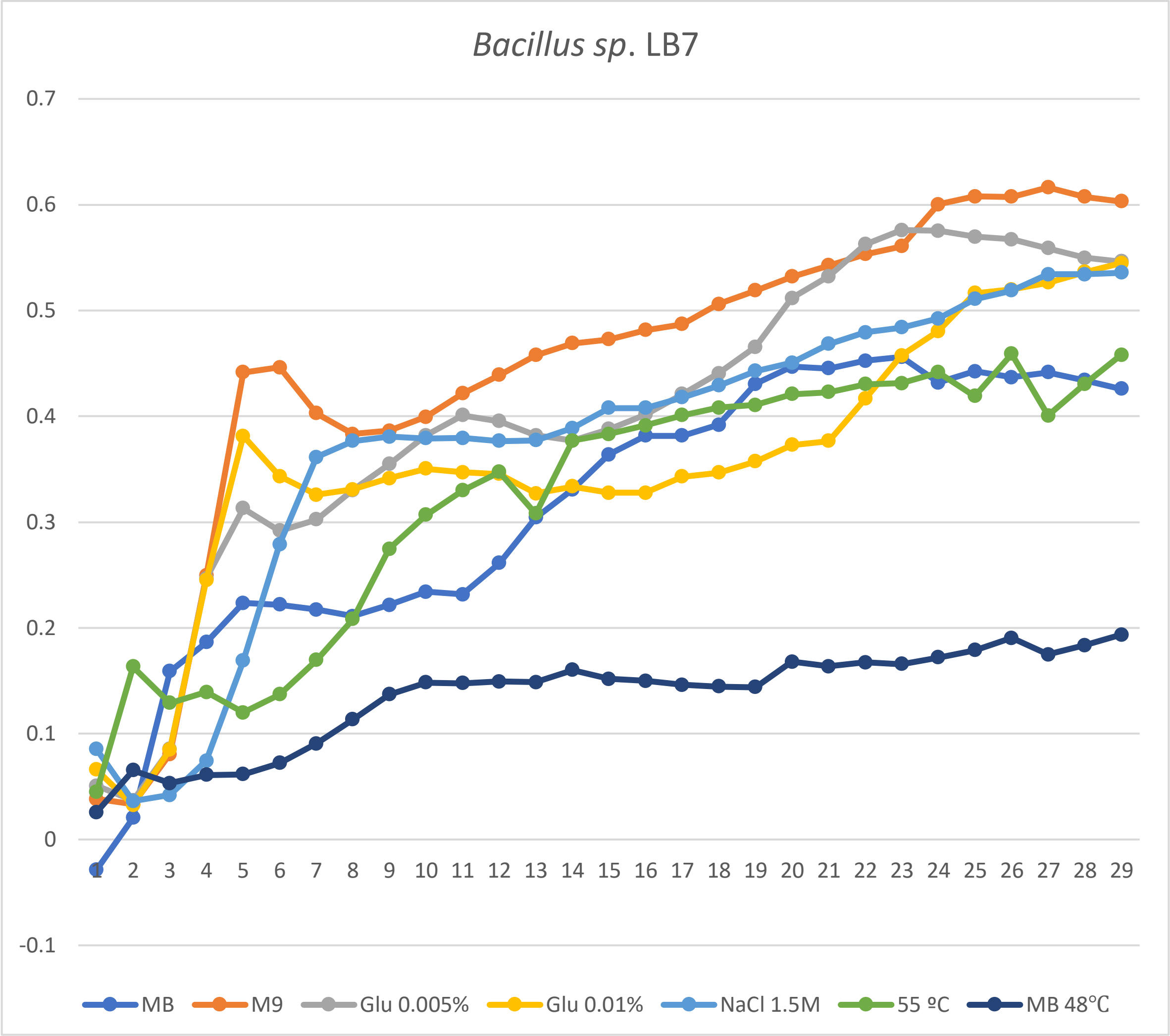
Bacillus sp. LB7
0.7
0.6
0.5
0.4
0.3
0.2
0.1
0
-0.1
1 2 3 4 5 6 7 8 9 10 11 12 13 14 15 16 17 18 19 20 21 22 23 24 25 26 27 28 29
MB
M9
Glu 0.005%
Glu 0.01%
NaCl 1.5M
55 ºC
MB 48°C

B)

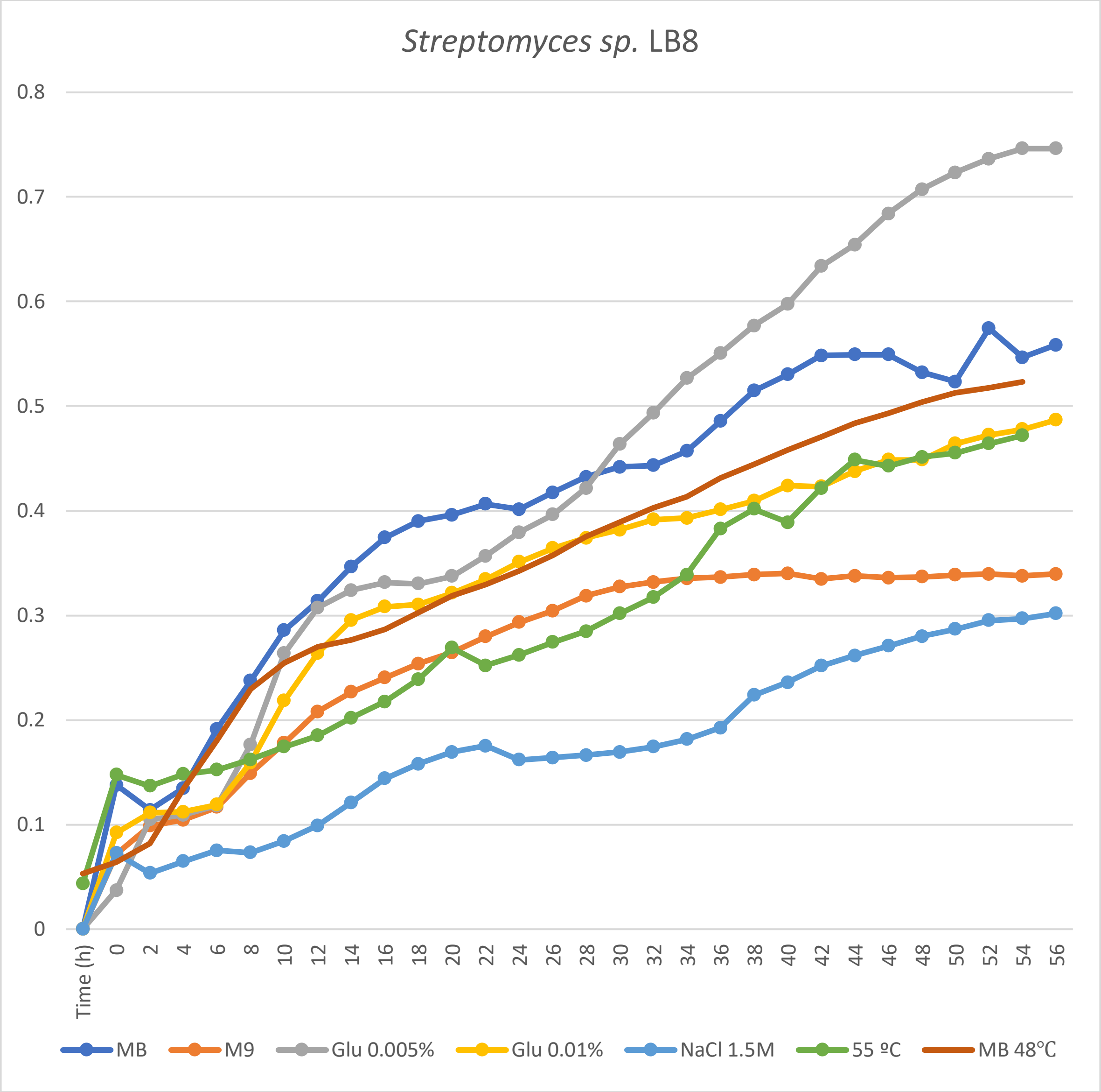

Supplementary Figure S2. Growth curve of LB7 and LB8 at different culture conditions. A) LB7 strain grown in M9 medium and MB, MB supplemented with Glucose 0.005% and 0.01%, NaCl 1.5 M and at 48 °C and 55 °C B) LB8 strain grown in M9 medium and MB, MB supplemented with Glucose 0.005% and 0.01%, NaCl 1.5 M, at 48 °C and at 55 °C. Curves show an average of 5 replicates.
