# Supplementary material for: Secondary metabolites with antimicrobial activity produced by thermophilic bacteria from a high-altitude hydrothermal system
Source: Front Microbiol. 2024 Sep 30;15:1477458. doi: 10.3389/fmicb.2024.1477458 (PMC11474921; doi:10.3389/fmicb.2024.1477458)
Supplement: Supplementary file 3 [file Data_Sheet_3.PDF]

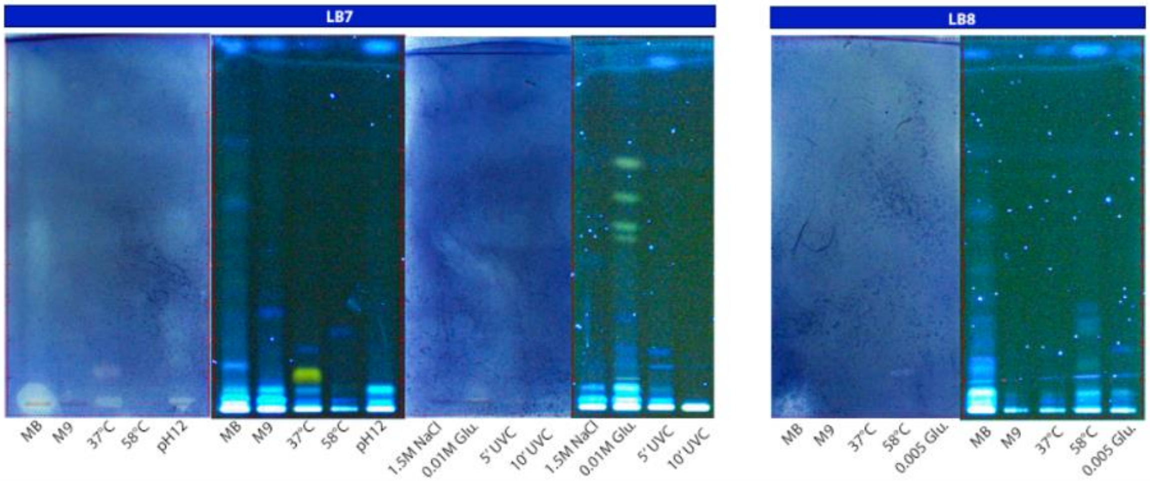

Supplementary Figure S3. Antimicrobial activity of the extracts obtained from LB7 and LB8 under different physicochemical conditions. The absence of coloration denotes positive results for antimicrobial activity. The left panels show the HPTLC plate revealed in visible light; while right panels show the plate at 336 nm for identifying bands before the antibacterial assay with *Bacillus subtilis*.
