# Supplementary material for: Secondary metabolites with antimicrobial activity produced by thermophilic bacteria from a high-altitude hydrothermal system
Source: Front Microbiol. 2024 Sep 30;15:1477458. doi: 10.3389/fmicb.2024.1477458 (PMC11474921; doi:10.3389/fmicb.2024.1477458)
Supplement: Supplementary file 4 [file Data_Sheet_4.PDF]

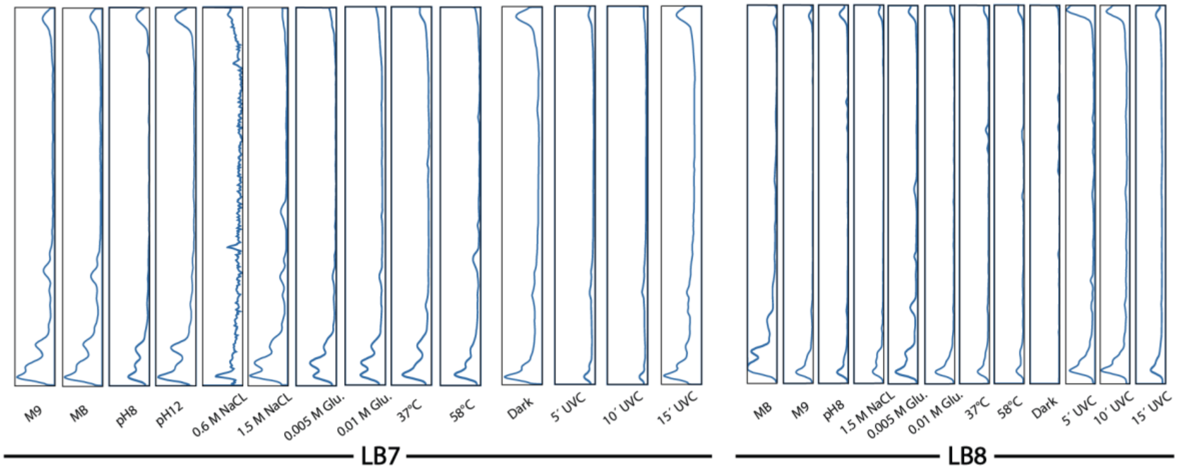

Supplementary Figure S4. Intensity plot representing the chromatograms band profile of the organic extract obtained from LB7 and LB8 strains. Each peak represents the corresponding band in the gel.
