# Supplementary material for: Secondary metabolites with antimicrobial activity produced by thermophilic bacteria from a high-altitude hydrothermal system
Source: Front Microbiol. 2024 Sep 30;15:1477458. doi: 10.3389/fmicb.2024.1477458 (PMC11474921; doi:10.3389/fmicb.2024.1477458)
Supplement: Supplementary file 5 [file Table_1.DOCX]

| **Supplementary Table S1. Physicochemical parameters obtained *in situ* in the hydrothermal pond.** | | | | | | |
| --- | --- | --- | --- | --- | --- | --- |
| **Sample** | **Temperature (°C)** | **pH** | **Salinity (psu)** | **Turbidity (FNU)** | **Conductivity (IS/cm^3^)** | **Redox potential (mV)** |
| **P42** | 42-48 | 7.5 | 0.89 | 4.5 | 1,837 | -152.4 |
