# Supplementary material for: Secondary metabolites with antimicrobial activity produced by thermophilic bacteria from a high-altitude hydrothermal system
Source: Front Microbiol. 2024 Sep 30;15:1477458. doi: 10.3389/fmicb.2024.1477458 (PMC11474921; doi:10.3389/fmicb.2024.1477458)
Supplement: Supplementary file 6 [file Table_2.docx]

| Supplementary Table 2. Identification of compounds obtained from LB7 by the mass spectrometer analysis, based on the Metabolic In Silico Network Expansions database. MINE code corresponds to the KEGG codification | | | | | | |
| --- | --- | --- | --- | --- | --- | --- |
| **Strain** | **Band** | **m/z** | **Identification (MINE)** | **MINE code** | **Nature** | **Associated function** |
| **LB7 grown in**  **MB** | A | 101.2 | Unknown | - | - | - |
|  | A | 135.5 | Unknown | - | - | - |
|  | A | 182.05 | Unknown | - | - | - |
|  | B | 336.05 | Mersalyl acid by-product (KEGG) | 661414 | Carbolixic acid | Metabolism |
|  | B | 341.25 | 2-octaprenyl phenol by-product (KEGG) | 2640 | Phenol | Metabolism |
|  | B | 404 | GSSeH by-product  (KEGG) | 563073 | Thioselenide | Selenium degradation |
|  | C | 267.2 | Unknown | - | - | - |
|  | C | 268.25 | Unknown | - | - | - |
|  | D | 244.3 | Unknown | - | - | - |
|  | D | 290.25 | Procyclidine by-product | 600739 | Procyclidine | Anticholinergic agent |
|  | D | 314.25 | (2E)-Hexadecenoyl-CoA by-product | 19472 | Acidanhydrine  acyclicphosphorus | Catobolization of  dimethylsulfoniopropionate |
|  | D | 360.3 | Unknown | - | - | - |
| **LB7 grown with**  **0,01M Glucose** | A | 101.2 | Unknown | - | - | - |
|  | A | 135.2 | Unknown | - | - | - |
|  | A | 186.05 | Unknown | - | - | - |
|  | A | 232.05 | Phosphophosphinate by-product | 566860 | - | Analogue inhibitor for D-Ala-D-Ala,  vancomicin resistance |
|  | A | 234 | Unknown | - | - | - |
|  | B | 275.2 | Myxalamide C by-product | 141593 | - | Antibacterial |
|  | B | 336.05 | Mersalyl acid by-product (KEGG) | 661414 | Carboxilix acid | Metabolism |
|  | B | 404 | GSSeH by-product  (KEGG) | 567316 | Thioselenide | Selenium degradation |
|  | B | 415.35 | Unknown | - | - | - |
|  | | | | | | |
| **LB7 grown at**  **pH12** | A | 135.2 | Unknown | - | - | - |
|  | A | 192.1 | 4-guanidinobutanoate by-product (KEGG) | 144627 | Butanoic Acid | Glutamate synthesis |
|  | A | 193.15 | Unknown | - | - | - |
|  | A | 265 | Dihydroxymandelic Acid  by-product | 94678 | Phenol | Antioxidant-metabolite of  norepinephrine |
| **LB7 grown with**  **1,5M NaCL** | A | 101.2 | Unknown | - | - | - |
|  | A | 135.2 | Unknown | - | - | - |
|  | A | 186.05 | Unknown | - | - | - |
|  | A | 208.1 | Trihomomethionine by-product | 164650 | Organic sulfur | Glucosinolate precursor |
|  | A | 223.05 | Imidazole-acetol phosphate by-product | 22677 | Azole | Histidine production |
|  | A | 226.1 | Amide biotine by-product | 261939 | Carboxamide | Antioxidant, antibacterial |
|  | A | 232.05 | Phosphophosphinate by-product | 566860 | Acid Anhydride  acyclic phosphorus | Chelator |
|  | A | 240.1 | Flavone by-product | 231654 | Flavonoid | Antioxidant, anti inflammatory,  anti-mutagenesis |
|  | A | 249.1 | Cryptolepine by-product | 569935 | Alkaloid | Cytotoxic, antimalarial |
| **LB7 grown**  **at 37°C** | A | 101.2 | Unknown | - | - | - |
|  | A | 134.75 | Unknown | - | - | - |
|  | A | 155.15 | Unknown | - | - | - |
|  | A | 156.15 | Unknown | - | - | - |
|  | A | 186.05 | Unknown | - | - | - |
|  | A | 208.1 | Trihomomethionine by-product | 164650 | Organic sulfur | Glucosinolate precursor |
|  | A | 223.05 | Amide bioteine by-product | 22677 | Azole | Histidine precursor |
|  | A | 232.05 | Phospho Phosphinate by product | 566860 | Acid Anhydride  acyclic phosphorus | Chelator |
|  | A | 234 | Unknown | - | - | - |
|  | A | 236.05 | Thiabendazole by-product | 481409 | Thiazole | Anti-fungal |
|  | A | 240.1 | Flavone by-product | 231654 | Flavonoid | Antioxidant, anti inflammatory,  anti-mutagenesis |
|  | A | 249.1 | Cryptolepine by-product | 569935 | Alkaloid | Cytotoxic, antimalarial |
|  | B | 276.15 | 2-phenyl-1(1,2,4-triazol-1-yl)-3-trimethylsilyl  propan-2-ol | 478904 | Thiazole | Antibacterial and antifungal |
|  | B | 336.05 | By-product of Mersalilic Acid (KEGG) | 661414 | Carboxylic acid | Metabolism |
|  | B | 430.95 | Unknown | - | - | - |
|  | B | 554.6 | Unknown | - | - | - |
|  | C | 155.2 | Unknown | - | - | - |
|  | C | 457.3 | Unknown | - | - | - |
