# Supplementary material for: Secondary metabolites with antimicrobial activity produced by thermophilic bacteria from a high-altitude hydrothermal system
Source: Front Microbiol. 2024 Sep 30;15:1477458. doi: 10.3389/fmicb.2024.1477458 (PMC11474921; doi:10.3389/fmicb.2024.1477458)
Supplement: Supplementary file 7 [file Table_3.docx]

| **Supplementary Table 3. Identification of compounds obtained from LB8 by the mass spectrometer analysis, based on the Metabolic In Silico Network Expansions database. MINE code corresponds to the KEGG codification** | | | | | | |
| --- | --- | --- | --- | --- | --- | --- |
| **Strain** | **Band** | **m/z** | **Identification (MINE)** | **MINE code** | **Nature** | **Associated function** |
| **LB8 grown in**  **MB** | A | 280.15 | 3-hexoxy-4-(1-methyl-2H-pyridin-3-yl)-1,2,5- thiadiazole | 361231 | Cholinergic | Associated to acetylcholine |
|  | B | 199.2 | Unknown | - | - | - |
|  | B | 212.2 | cis-11-metil-2-dodecenoicacid  by-product | 509867 | Fatty acids | Quorum sensing |
|  | B | 314.3 | Unknown | - | - | - |
|  | C | 122.15 | Unknown | - | - | - |
|  | C | 267.2 | Unknown | - | - | - |
|  | C | 269.25 | 12,13-Epoxy-9-hydroxy-10-octadecenoate by-product | 271144 | Octadecanoid | Defense |
|  | D | 118.2 | Unknown | - | - | - |
| **LB8 grown**  **at 58°C** | A | 183.2 | Unknown | - | - | - |
|  | A | 358.2 | Spiridine by-product | 144655 | Alkaloid | Radiation response |
|  | A | 385.3 | Unknown | - | - | - |
|  | A | 399.3 | Unknown | - | - | - |
|  | B | 199.2 | Unknown | - | - | - |
|  | B | 314.3 | Unknown | - | - | - |
|  | B | 355.3 | 15,9'-dicis-phytofluene by-product | 255133 | Isoprenoids | Carotenoid production, antioxidant |
|  | B | 356.3 | Unknown | - | - | - |
| **LB8 grown**  **at 37°C** | A | 399.3 | Unknown | - | - | - |
|  | A | 430.95 | Unknown | - | - | - |
|  | A | 498.9 | Unknown | - | - | - |
|  | A | 566.9 | Unknown | - | - | - |
|  | A | 634.95 | Unknown | - | - | - |
|  | B | 100.15 | Unknown | - | - | - |
|  | B | 115.15 | Octane | 123182 | Hydrocarbon | Hydrocarbon |
|  | B | 118.2 | Unknown | - | - | - |
|  | B | 122.15 | Unknown | - | - | - |
| **LB8 grown with**  **0,005M Glucose** | A | 355.95 | Unknown | - | - | - |
|  | A | 357.25 | Unknown | - | - | - |
|  | A | 362.25 | Aurachin by-product | 337935 | Alkaloid | Antibacterial |
|  | A | 399.3 | Unknown | - | - | - |
|  | A | 419.25 | 4-[5-[[4-[5-ammonium pentil(hydroxy)  amino]-4kketo-butanoyl]amino]pentyl hydroxy-amino]-keto-butyrate | 393157 | Siderophore | Bisucaberin by-product |
|  | A | 429.3 | Nuatigenin by-product | 574665 | Sterol | Metabolite |
|  | A | 430.95 | Unknown | - | - | - |
|  | A | 498.9 | Unknown | - | - | - |
|  | A | 566.9 | Unknown | - | - | - |
|  | A | 634.95 | Unknown |  |  |  |
|  | B | 100.15 | Unknown | - | - | - |
|  | B | 122.15 | Unknown | - | - | - |
|  | B | 135.2 | Unknown | - | - | - |
|  | B | 313.2 | By-product of Neomethymicinor10-  Deoxymetinolide | 158429 | PKS (Macrolides  and Lactone) | Antibacterial |
|  | B | 376.2 | Unknown | - | - | - |
|  | C | 100.15 | Unknown | - | - | - |
|  | C | 115.15 | Octane | 123182 | Hydrocarbon | Hydrocarbon |
|  | C | 312.2 | By-product of Neomethymicinor10-  Deoxymetinolide | 158429 | PKS (Macrolides  and Lactone) | Antibacterial |
|  | C | 340.1 | By-product of Pancratistatin | 439152 | Citraconoyl group | Anticancerigen |
| **LB8 grown at 48°C**  **after 5 UVC**  **pulses** | A | 100.15 | Unknown | - | - | - |
|  | A | 101.15 | Unknown | - | - | - |
|  | A | 115.15 | Octane | 123182 | Hydrocarbon | Hydrocarbon |
|  | A | 135.2 | Unknown | - | - | - |
